# Genetic Variation for Cardiac Dysfunction in *Drosophila*

**DOI:** 10.1371/journal.pone.0000601

**Published:** 2007-07-11

**Authors:** Karen A. Ocorr, Timothy Crawley, Greg Gibson, Rolf Bodmer

**Affiliations:** 1 Center for Neuroscience and Aging, The Burnham Institute for Medical Research, La Jolla, California, United States of America; 2 Department of Genetics, North Carolina State University, Raleigh, North Carolina, United States of America; University of Chicago, United States of America

## Abstract

**Background:**

Common diseases may be attributed to combinations of variant alleles, but there are few model systems where the interactions among such variants can be studied in controlled genetic crosses. While association studies are designed to detect common polymorphisms of moderate effect, new approaches are required to characterize the impact on disease of interactions among rare alleles.

**Methodology/Principal Findings:**

We show that wild populations of *Drosophila melanogaster* harbor rare polymorphisms of major effect (RAME) that predispose flies to a specific disease phenotype, age-dependent cardiac dysfunction. A screen of fifty inbred wild-type lines revealed a continuous spectrum of pacing-induced heart failure that generally increases in frequency with age. High-speed video analysis of the inbred lines with high rates of inducible heart failure indicates specific defects in cardiac function, including arrhythmias and contractile disorders (‘cardiomyopathies’). A combination of bulked segregant analysis and single feature polymorphism (SFP) detection localizes one of the cardiac susceptibility loci to the 97C interval on the fly genome.

**Conclusions/Significance:**

Wild-type *Drosophila*, like humans, are predisposed to cardiac dysfunction. Identification of factors associated with these naturally occurring cardiac traits promises to provide important insights into the epidemiology of cardiac disease.

## Introduction

*Drosophila melanogaster* has been widely adopted as a model organism for investigating the function of biomedically relevant genes [1], [2], but to date has been underutilized in investigations of the genetic basis of complex diseases. By contrast, quantitative genetic studies of *Drosophila* have focused on non-medical traits such as bristle number, wing shape, and aspects of life history including longevity and fecundity [3], [4]. To our knowledge, no studies have previously attempted a genetic characterization of an actual hereditary disease or organ malfunction that afflicts otherwise normal flies in the wild. Since cardiovascular disease is the greatest cause of mortality in contemporary humans, we set out to ask whether wild-type flies might exhibit genetic variation for cardiac disease–related phenotypes, including heart failure and arrhythmias.

Pacing-induced cardiac dysfunction or failure is a rapid and efficient assay for detecting heart performance deficits in *Drosophila*. Application of external electrical stimulation elevates the normal heart rate for a short time, which may or may not result in heart failure (defined as fibrillation or arrest) at a rate that is characteristic for the genotype and age of the line of flies [5]. Typically, young flies rarely fail whereas older flies fail more often in response to this stress protocol. In addition, flies with defective insulin signaling exhibit a considerably reduced rate of cardiac failure at old age [6]. Previous and current screens of mutant lines have demonstrated that perturbation of ion channel physiology and cardiogenesis also impacts the incidence of heart failure [7]–[9; Dowse et al, 1995; K.O., L. Qian and R.B. unpublished]. Furthermore, KCNQ potassium channel mutations can cause arrhythmias in humans as well as in flies [8], and Optical Coherence Tomography provides evidence for severe impairment of systolic function due to expression of a mutant sarcoglycan that causes a similar phenotype, dilated cardiomyopathy, in flies and humans [10].

Such observations raise the possibility that polymorphism in a variety of genes might contribute to heart disease in natural populations [11], [12]. A standard model for common complex diseases is that common variants of moderate effect combine together to increase disease susceptibility [13], [14], though other models also need to be considered. Rare alleles of major effect, including complete loss of function mutations, individually are not expected to contribute measurably to risk in a population, but hundreds of recessive mutations each at a frequency of 0.01 could account for disease in a few percent of individuals. Deleterious interactions between such rare alleles in compound heterozygous carriers have the potential to account for a substantial public health burden. Unfortunately, genomewide association approaches have almost no power to detect RAME, so new approaches are needed in order to detect them and to establish their capacity for compound interactions.

In this paper we describe one such strategy in the context of cardiac failure in *Drosophila*. We show that wild populations of *D. melanogaster* indeed carry major-effect cardiac failure-promoting alleles, and that these can be mapped by standard linkage methods.

## Materials and Methods

### Pacing-induced heart failure assay

**Figure 1:**
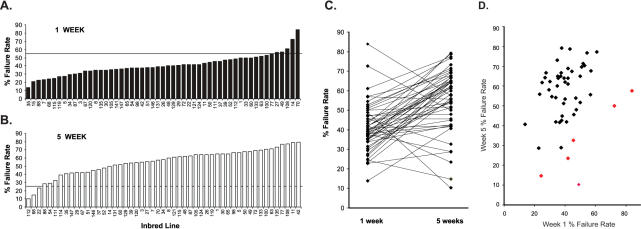
Cardiac failure rates in inbred wild-type lines. (A) Histogram of pacing-induced 1-week heart failure rates in 52 inbred lines. Lines are rank-ordered by failure rate in 1 week old flies. (B) Similarly for 5-week old flies. Cutoff lines show 55% and 25% failure rates, respectively. (C) The two measures are significantly correlated (p<0.01) for all but six lines that account for most of the extreme effects, as shown by the crossing of line means between 1 week (left) and 5 week (right) failure rates. (D) Correlation between failure rates at the two ages, with the six extreme lines shown in red.

Pacing-induced heart failure was performed as described in [5], [6]. Briefly, for at least 50 adult flies of each age, in groups of 4–8 per vial, an attempt was made to induce heart failure by stimulating the dorsal abdomen at 40V and 6 Hz for 30s using two electrodes smeared with a conductive jelly to make contact with the head and the tail of the fly. The heart failure rate in Figure 1 is defined as the percentage of flies that either enter fibrillation or undergo cardiac arrest during or immediately after the stimulation. All failure rates are shown in Text S1. Significance of line differences was established using a non-parametric Chi-squared test, and confirmed by replication on samples several months apart (see Text S2).

### Image analysis of rhythmicity in semi-intact preparations

**Figure 2:**
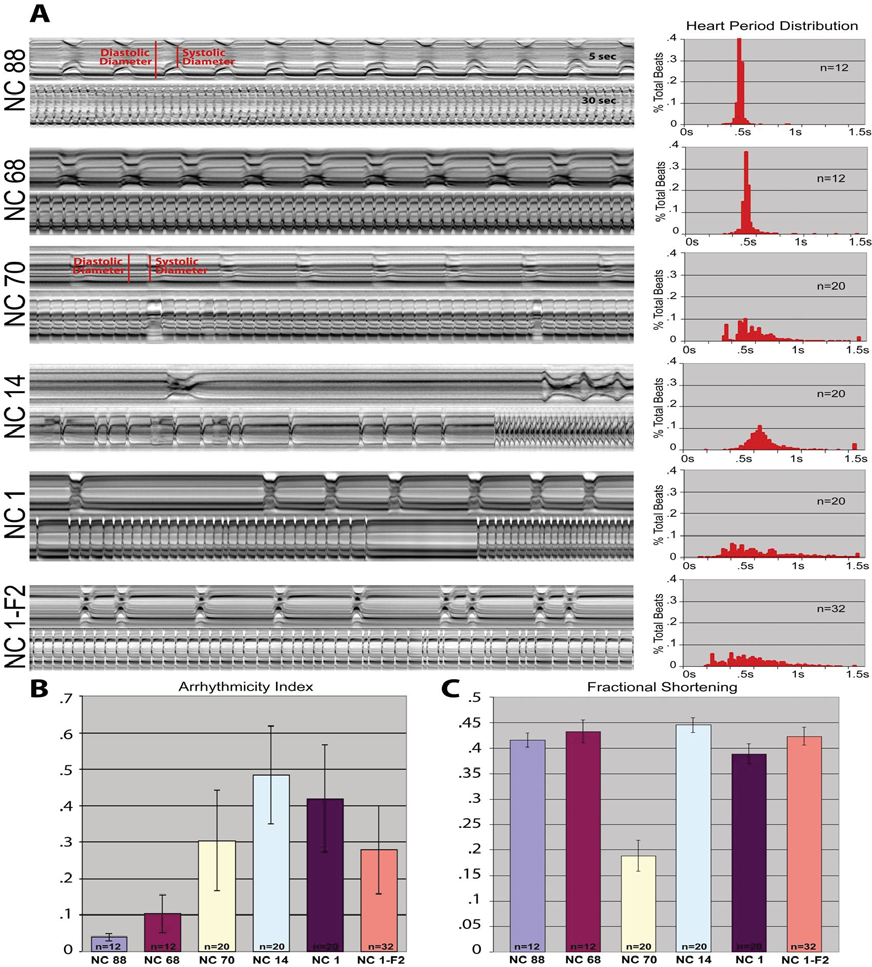
Visualization of arrhythmicity in 1 week old flies. Each panel shows a 30 second M-mode profile underneath an expanded 5-second profile. Lines NC88 and NC68 have normal rhythmicity, and lines 70, 14 and 1 show different modes of arrhythmia. A representative F2 progeny of the *yw* × NC1 cross is also shown. Histograms to the right of each profile indicate the distribution of beat lengths (heart period) for all flies in each genotype. Data were normalized to the average of the median values and expressed as a percentage of the total beats recorded. Heart periods longer than 1.5s were grouped together and are shown in the final bar of the histograms (they can be considered as ‘asystoles’). (B) Heart beat rhythmicity was quantified as the standard deviation of heart period, which clearly shows the increased variance of beat length in the arrhythmic lines. (C) Fractional shortening, computed as the ratio of the systolic to the diastolic diameters (see red lines in panel A), is a measure of the ejection volume, which is significantly reduced in NC70 relative to that in NC88.

M-mode traces documenting the movement of the edges of the heart tube were performed as described in [8]. Flies were anaesthetized for 2–5 minutes with FlyNap, their heads and ventral thorax were removed, and the abdominal dorsal vessel was exposed by carefully dissecting away ventral tissues and fat under oxygenated artificial hemolymph. Such preparations typically beat rhythmically for several hours, but all measurements were made after 20 minutes of equilibration at room temperature. High speed 20 second movies were taken at a rate of >100 frames per second using a Hamamatsu EM-CCD digital camera on a Leica DM LFSA microscope with a 10x dipping immersion lens (See Video S1 and Video S2 for normal and abnormal examples respectively). The images were processed using Simple PCI imaging software (Compix, Inc, Lake Oswego OR), and M-modes were generated using custom MatLab code written by Martin Fink (Oxford University) that extracts a single pixel wide column from the same location in each successive frame of the movie, and aligns them horizontally into a sequential trace with time along the x-axis and movement of the heart tube on the y-axis (as in [8]). Automated digital contrast feature extraction was then used to compute quantitative data shown in Figure 2, including beat length histograms and mean fractional shortening (the ratio of the diameter of the heart edge at systole and diastole).

### Bulked Segregant Analysis of NC1

Low resolution mapping of the genetic region that explains a large proportion of the 1 week heart failure phenotype in the NC1 line was performed using Single Feature Polymorphism (SFP) analysis of Bulked Segregant pools of F2 flies from the cross with male tester yw flies. Raw array data is available online as the Text S3 file. SFP was performed using a modification of a protocol supplied by S. Nuzhdin (UC Davis), essentially following the approach of J. Borevitz [15]. Genomic DNA was extracted from 15 – 20 individuals each of the NC1 isogenic line, a tester strain (yw), and F2 adults that exhibit cardiac failure. No attempt was made to enrich for the most severe failure since affected individuals generally have the same defect: the quantitative aspect of the trait is the penetrance of a threshold-dependent defect, rather than expressivity. Since cardiac failure was observed in approximately one half of the individuals scored, we inferred that a partially penetrant dominant allele was likely segregating in the cross. This leads to the expectation that in regions linked to the trait, up to two thirds of the flies should be heterozygotes and the remaining one third should be homozygous NC1, resulting in 67% of the alleles being derived from the NC1 parent. If the allelic effect is only partially dominant, the fraction of NC1 alleles will increase accordingly. SFP analysis was performed using a whole genome amplification of each DNA pool, digestion to approximately 200 bp fragments, labeling with biotin, and hybridization to Drosophila 2.0 Affymetrix GeneChips (performed by Expression Analysis Inc, Durham NC). Two replicate F2 samples were scored. After standard normalization of perfect match probes to the array mean, over 90% of the probes are within 1.25-fold of one another in hybridization intensity for the two parents, and these were discarded, leaving probes that are potentially mis-hybridizing to either genome due to a sequence polymorphism.

**Figure 3:**
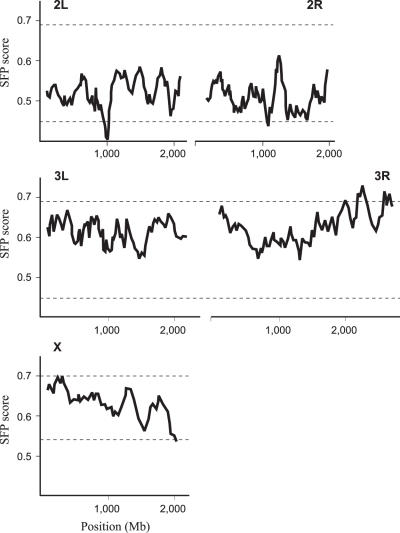
Linkage of NC1 defect to interval 97C. The plot of SFP score according to chromosomal position shows a clear peak toward the end of chromosome 3R with a maximum at interval 97C (the second peak at the tip of 3R was only seen in the second replicate). A second peak in the *yw* region at the tip of the X chromosome is also seen. Dashed lines show 99% permutation limits for sliding window analysis of scores.

Probes with greater deviation than this between NC1 and *yw* were selected as candidate SFPs, consistent with expectations given the high level of DNA sequence polymorphism in Drosophila. We then asked whether the F2 hybridization intensity for each probe was closer to the NC1 or *yw* intensity by computing the value of |yw–F2|/|yw–NC1|. A value of 0 implies that the *yw* genotype dominates the pool; 1 implies that NC1-derived alleles are prevalent; and 0.5 implies an equal mix. Absolute values greater than one (approximately 1% of the remaining probes) were excluded from further analysis as they do not make biological sense and presumably represent measurement error. We then plotted a sliding window along each chromosome arm of the average SFP score over 100 SFPs, as shown in Figure 3, which shows the mean of the two replicates. 100 permutations of SFP positions were used to set the 95% confidence limit on extreme values for the autosomes and the X-chromosome separately. The indicated peak at 97C was greater than all permutation values and was observed in both replicates, as was the peak near the tip of the X-chromosome in the vicinity of *yw*. A dip in the profile in the middle of chromosome arm 2L may indicate a suppressor of heart failure in the NC1 line, but it was only significant in the second replicate.

## Results and Discussion

### Survey of wild-type lines for age-dependent cardiac dysfunction

The incidence of pacing-induced heart failure was measured in one-week and five-week old flies from each of 50 highly inbred wild-type lines (30–100 flies per line) derived from a North Carolina peach orchard [16], [17]. In most young wild-caught fly lines (as well as in laboratory ‘wildtype’ lines), 20–45% of tested individuals experience heart failure upon pacing, but with age the proportion increases to 50–80% (Figure 1A,B). Two lines (out of 50) exhibit a much elevated failure rate exceeding 70% at 1 week of age (lines 14 and 70). By contrast, six lines are unusually resistant to heart failure at old age, most notably lines 112 and 68, each with failure rates less than 15%. The black bars on the histogram in Figure 1A indicate the rank order of 1-week failure rates, while the white bars on the bottom panel 1B show the corresponding failure rates at 5 weeks. Across all 50 lines measured at both ages, only around five percent of the total variation is explained by line differences, and hence genetic variation. However, the most extreme line effects are highly replicable (see supplemental data), suggesting the segregation of genetic differences in these lines. Nominal logistic regression indicates that the line, age, and line-by-age interaction effects are all highly significant (*p*<0.0001, Figure 1C), but six lines show an opposite trend, ie. reduced failure rate with age. These six lines are the two hyper-sensitive young lines, and four of the five hyper-resistant lines at old age. Excluding these six lines, the genetic correlation between the two stages of life is greater than 0.45, indicating that pacing-inducible heart failure tends to increase with age to a similar degree in the majority of lines, as previously reported for laboratory ‘wildtype’ flies [5]. However, the six extreme lines define mutants that appear to reverse the trend of age-dependence for rate of pacing-induced failure, indicating that high heart failure is not simply due to fixation of generically deleterious alleles during inbreeding of the lines.

### Visualization of cardiac defects by high resolution imaging

Ten lines with elevated heart failure rate and several with moderate or low failure rate were then analyzed in more detail for visible defects in heart performance. Adult hearts of up to 24 individuals of each line were exposed by dissection in oxygenated saline, allowing the heart beat to be captured with a high-speed camera at 100–200 frames per second (see [8], materials and methods and supplemental movie). An optical section one pixel wide at a common location (middle of the third abdominal segment) was taken and these were aligned temporally along the horizontal axis to produce M-mode traces (Figure 2A). Changes in heart rate, diameter of the heart tube, ejection volume, duration of contractions and inter-beat intervals, and importantly, changes in various aspects of heartbeat rhythm, were studied by monitoring fluctuation in optical density, which is associated with movement of the heart walls, and by tracking changes in individual pixel intensities.

In dissected individuals of most lines and in laboratory ‘wildtype’ animals, the young adult heartbeat is highly rhythmic, with a constant ejection volume, systolic and diastolic (interbeat) interval length (lines NC88 and 68 in Fig. 2A,C; [8]). In sharp contrast, representative M-mode traces of the two lines that are most susceptible to failure, lines NC14 and NC70, show considerable aberrations from the normal rhythmicity profiles. Semi-automated measurement of the length of the heart periods confirms that these aberrant lines have greatly increased variability of heartbeat lengths (Figure 2A, histograms on the right), the standard deviation of which provides a quantitative measure of arrhythmia (Figure 2B). In addition, the NC70 line exhibits a very shallow contraction profile reminiscent of patients with dilated cardiomyopathy, as indicated by an unusually small fractional shortening (systolic/diastolic diameter; Figure 2C). A similar dilated phenotype has previously been observed by expressing a human cardiac disease mutation in flies [10]. It is remarkable that each of the five most pacing-sensitive lines show clear evidence for aberrant heart rhythm in over half of the young adults (Table 1). This suggests that among individuals of wild fly populations there are some that exhibit disease–related phenotypes, including elevated pacing-inducible heart failure, arrhythmias and cardiomyopathies.

**Table 1:** Number of Aberrant Heart M-Mode Profiles by Line and Cross.

	NC70	NC14	NC108	NC49	NC27	NC100	NC63	NC60	NC33	NC1	NC22
Homozygous %	11/14	13/19	16/19	14/24	9/20	0/5	0/6	1/6	1/6	11/18	0/10
	78	68	84	58	45	0	0	17	17	61	0
*yw* Outcross %	9/18	9/23	13/22	8/21	4/18	-	-	-	-	13/25	-
	50	39	59	38	22	-	-	-	-	52	-

### Genetic analysis suggests a simple basis for certain defects

Next, genetic crosses were performed to ascertain whether the disease phenotype in the wild-type lines has a simple or polygenic basis. Between 18 and 25 progeny of a cross between each sensitive line and a standard laboratory strain, *yw*, were scored, and six lines with aberrant heart beats were also aberrant when outcrossed, suggesting a partially dominant mode of transmission. Approximately one half of the F2 progeny of one of the lines, NC1, crossed to a normal (*yw*) strain, exhibit defects that are remarkably similar to those observed in NC1 homozygotes (Figure 2A–C), suggesting a clearly dominant monogenic mode of transmission.

The hypothesis that segregating major effect alleles account for a large portion of the disease phenotype in the NC1 line was confirmed by performing linkage mapping of the NC1 defect, using Single Feature Polymorphism (SFP) detection in conjunction with Bulked Segregant Analysis (BSA) [15], [18] (see Figure 3 and materials and methods for details). Two replicate samples of genomic DNA each extracted from 10 F2 flies displaying heart failure in the testcross of NC1 by *yw* resulted in a highly significant linkage peak at the identical position on the right arm of chromosome 3 (Figure 3), consistent with the presence of a dominant susceptibility locus centered on the interval 97C in the NC1 line.

Further evidence for linkage of the 97C region to cardiac failure in the NC1 cross was obtained by sequencing 34 random clones derived from the two independent pools of F2 genomic DNA, encoding a 500 bp fragment of the *beat-VII* locus, CG14249. Two sequence polymorphisms were detected, and for both of these, 25 clones derived from the NC1 strain allele, and 9 from the *yw* strain allele. This proportion, 73% NC1, is the same as that observed in the SFP analysis. Assuming incomplete penetrance for a purely dominant susceptibility allele in affected individuals, two thirds of the alleles would be expected to be from the NC1 parent. However, since NC1 homozygotes show slightly higher incidence of young adult heart failure than outcrossed heterozygotes (61% versus 52%, Table 1), the observed fraction of 73% NC1 alleles is consistent with the expected segregation ratio if the 97C allele accounts for the majority of the genetic defect. Confirmation of the linkage peak in an independent sample of F2 flies is under way, and genotyping of individual affected and unaffected individuals will establish the relative risk of disease due to the 97C allele in NC1. Introgression experiments involving this locus as well as peaks indentified in other crosses, are planned in order to estimate the magnitude of effect of the polymorphisms across a range of genetic backgrounds, as well as to monitor their capacity for heterozygous interaction.

According to FlyBase, *beat-VII* encodes a calcium-binding protein and is a member of an immunoglobulin superfamily that is implicated in axon guidance, but there is little genetic or biochemical data available to confirm a function in heart physiology. None of the dozen other genes in the vicinity are annotated, so further fine structure mapping and molecular genetic analyses will be required to establish the identity of the gene that causes the cardiac failure.

These results suggest that wild-type flies suffer from age-dependent heart failure, and that this disease is likely to be genetically dissectible. The process of inbreeding concentrates alleles and focuses their effects in homozygotes, so the observed incidence of disease in fifteen percent of lines is likely greater than that seen in outbred flies. On the other hand, if hundreds of major-effect heart disease susceptibility alleles segregate in natural populations, their cumulative impact on cardiac failure both as homozygotes and compound heterozygotes could be substantial. Our experiments suggest a general strategy for testing this hypothesis by mapping rare alleles of major effect and measuring their effects on disease susceptibility in diverse genetic backgrounds and allelic combinations.

## Supporting Information

Text S1 Cardiac failure rates at 1 and 5 weeks of age. The three lefthand columns show whether or not each individual fly was normal or failed the pacing test. The four right hand columns summarize the data for both timepoints and express the difference as a percentage of the average failure rate at both times.
(0.41 MB XLS)

Text S2 Repeatability of failure rate measurements. This word file shows the repeatability of the failure rate estimates on samples measured several months apart.
(0.03 MB DOC)

Text S3 Raw data for SFP analysis. This text file shows the raw probe intensities for each of six Affymetric SPF arrays. Probesets are listed by probe number, and data is shown in respective columns for NC01, F1 of NC70xyw, yw, NC70, and two replicate crosses of NC01xyw. Note that only the NC01 mapping is described in the manuscript.
(11.63 MB TXT)

Video S1 Normal heart beat. An MP4 video file showing a normal one week old beating heart.
(1.95 MB MOV)

Video S2 Abnormal heart beat. This is an MP4 video showing an example of an abnormal one week old heartbeat from line NC014.
(0.65 MB MOV)
