# Supplementary material for: Genetic Variation for Cardiac Dysfunction in Drosophila
Source: PLoS One. 2007 Jul 11;2(7):e601. doi: 10.1371/journal.pone.0000601 (PMC1913553; doi:10.1371/journal.pone.0000601)
Supplement: Text S2 — Repeatability of failure rate measurements. This word file shows the repeatability of the failure rate estimates on samples measured several months apart. (0.03 MB DOC) [file pone.0000601.s002.doc]

**Replicability of Pacing Induced Failure**

The repeatability of the pacing-induced failure rate is established by the following data from assays conducted at least 5 months apart.

1 week Pacing

NC001 Rep 1 34/52 = 65%

NC001 Rep 2 22/48 = 46%

NC037 Rep 1 13/30 = 45%

NC037 Rep 2 19/40 = 48%

NC049 Rep 1 32/53 = 60%

NC049 Rep 2 24/46 = 52%

NC070 Rep 1 37/44 = 84%

NC070 Rep 2 37/44 = 84%

5 week Pacing

NC051 Rep 1 19/48 = 40%

NC051 Rep 2 13/27 = 44%

NC068 Rep 1 6/37 = 16%

NC068 Rep 2 19/68 = 28%

NC070 Rep 1 24/43 = 56%

NC070 Rep 2 14/23 = 61%

NC121 Rep 1 15/30 = 50%

NC121 Rep 2 22/29 = 76%

Nominal Logistic Regression on Line and Rep(Line) implies Line effects are highly significant (Chi-square LR = 84.7; p = 1E-015), while the Replicate effects are non-significant (Chi-square LR = 11.6; p = 0.17). The same result is obtained for each time considered separately. Note the repeatability of the time-dependence of the failure rate for line NC070.
